# Supplementary material for: Genome annotation across species using deep convolutional neural networks
Source: PeerJ Comput Sci. 2020 Jun 15;6:e278. doi: 10.7717/peerj-cs.278 (PMC7924482; doi:10.7717/peerj-cs.278)
Supplement: Supplemental Information 1 [file peerj-cs-06-278-s001.pdf]

1 SUPPLEMENTARY METHODS

Hyperparameter tuning

Samples of annotated sequences are divided into training (70%), test (15%) and validation (15%) sets. The training set is used to find the optimal weights using the back-propagation algorithm by pairing the input with expected output. The validation set helps to tune the hyperparameters and provides information about how well the model has been trained. It returns the model performance scores for each epoch and is used to determine a stopping point for the back-propagation algorithm in order to avoid overfitting. The test set helps to assess the quality of the fully-trained model over unseen samples.

To investigate the impact of different architectures of our CNN model on the network performance quality, the hyperparameters (number and size of layers, kernel size s, max-pooling window size m) of the model are tuned. Table 1 displays the impact of varying s, m and network depth on validation set across the balanced data with regard to accuracy score. As reported in the table, performance of different architectures did not vary significantly from each other. However, the architecture related to the highest accuracy score is selected as hyperparameters of our final CNN model (see Table 1).

Table 1. Results on hyperparameters tuning. The impact of different combinations of kernel size s, pool size m and network depth on accuracy score over validation set of the balanced data. The first column represents different kernel sizes for 3 convolutional layers. The second column shows different pool sizes used after each convolutional layer. The third column corresponds to the depth of the network. s is capable of recognizing relevant patterns within local neighborhood and m reduces input patterns to a lower dimension by combining important representations within the region. The first row values representing the highest accuracy score are selected as hyperparameters of the CNN model.

Kernel size	Pool size	Depth	Accuracy
(4x4, 4x4, 4x4)	(1x2, 1x2, 1x2)	3	0.829
(3x4, 4x4, 2x4)	(2x2, 2x2, 2x2)	3	0.807
(2x4, 3x4, 4x4,4 x4)	(1x2, 1x3, 1x2, 1x2)	4	0.791
(1x4, 1x4, 1x4, 1x4)	(1x2, 1x2, 1x2, 1x2)	4	0.812
(3x4, 4x4, 2x4, 4x4, 4x4)	(2x2, 1x2, 1x2, 2x2, 1x2)	5	0.802
(2x4, 2x3, 2x4, 1x4, 4x4)	(1x2, 1x2, 1x2, 1x2, 1x2)	5	0.785

1.1 Conventional performance measures

Several conventional measures are used to evaluate the performance of the CNN models. The proposed predictive models can be thought of a binary classifier by assigning a decisive threshold over their probabilistic outputs. Traditional measures for binary classification task are precision, recall, F1-score and accuracy. We define true positives (TP) as correctly predicted GSS, false positives (FP) as non-GSS wrongly classified as GSS, false negative (FN) as GSS wrongly classified as non-GSS and true negative (TN) as non-GSS correctly classified as non-GSS.

Precision is the ratio of correctly predicted GSS to the total predicted positive observations: $Pr = \frac{TP}{TP+FP}$. Thus, the high precision relates to the low false positive rate. Recall (or sensitivity) is the ratio of correctly predicted GSS to the all observations in positive class: $Re = \frac{TP}{TP+FN}$. F1-score is the weighted average of precision and recall, which takes equally both false positives and false negatives into account: F1-score $= 2 \times \frac{Re \times Pr}{Re+Pr}$. Specificity is the true negative rate or the proportion of non-GSS that are correctly identified $Sp = \frac{TN}{TN+FP}$. Accuracy is the most intuitive performance measure and it is defined by a ratio of correctly predicted observations (true positives and true negatives) to the total positive and negative observations: Accuracy = $\frac{TP+TN}{TP+FP+FN+TN}$. However, accuracy is not a reliable measure to assess model performance for datasets with unevenly distributed classes such as the unequal proportion of GSS and non-GSS samples. F1-score measure is usually more adequate than accuracy in uneven class distribution. Matthews Correlation Coefficient (MCC) is also used in bioinformatics as a performance metric and is often more reliable than the other measures for unbalanced data [**?**]. This measure takes into account the unbalance in the classes (in binary classification) and is defined as $MCC = \frac{TP \times TN - FP \times FN}{\sqrt{(TP+FP)(TP+FN)(TN+FP)(TN+FN)}}$. The MCC is a correlation coefficient value between -1 and $+1$, where a coefficient of +1 signifies an ideal prediction, -1 an inverse prediction and 0 an average

random prediction.

2 SUPPLEMENTARY RESULTS

2.1 Conventional measures for model performance assessment do not reflect genome-wide performances

While we focus in this paper on applying our trained models on full chromosome sequences with a sliding window, we also performed a more conventional machine learning analysis for the sake of completeness. To compare conventional performance metrics explained in 1.1 with our λ score, we evaluate the performance of all Q^* models (with $Q = 1, 10, 20, 30, 50, 70, 100$). To evaluate these models, we split each dataset in training (70%), test (15%) and validation (15%) sets. As reported in Figure Supplementary Figure 3, the CNN model applied on the balanced data (1*) yields the best performance on the test set regarding the precision/recall curve (PR) with respect to other Q^* models. Counter intuitively, the model giving the best scores on a conventional test set yields the poorest predictions when applied on the genome-wide scale.

Following this observation, we verify whether this holds also for other metrics commonly used to evaluate the performance of the CNN models over test sets. Supplementary Figure 4a recapitulates the results presented in Supplementary Figure 3. The Area Under Precision/Recall Curve (AUPRC) reveals an uppermost score for the balanced dataset but it deteriorates across the limited unbalanced datasets. The AUROC on the other hand presents stationary scores across all models. Given that there are many more true negatives than true positives within unbalanced datasets, PR is considered as a trustworthy measure because it does not take into account the true negatives. Indeed, AUPRC curve is misleading when applied to strongly unbalanced datasets, because the false positive rate (FP/total real negatives) does not decrease drastically when the total real negatives is huge. Whereas AUPRC is highly sensitive to FP, it is not impacted by a large total real negative denominator. In Supplementary Figure 4b, F1-score reports a weighted average between precision and recall per class. While, the F1-score enhances for non-GSS class across the datasets an opposite trend is observed for GSS class. This means that the more negative samples are introduced in the datasets, the more the model has the difficulty to return efficient predictions for GSS class. Figure Supplementary Figure 4c shows the scores for binary cross entropy, MCC and accuracy measures for all models. Binary Cross Entropy is the loss function that is used in this work the by back-propagation algorithm during training process. Cross entropy loss thus decreases as the predicted probability converges to the ground truth data. This metric improves when adding more negative examples into the balanced dataset, i.e. when Q increases. Regarding the accuracy score, it reaches its maximum for unbalanced datasets as well. In the unbalanced data scenario, accuracy is not any more a reliable measure. As a matter of fact, machine learning algorithms are usually designed to improve accuracy by reducing the error. Thus, facing unbalanced datasets, they produce inadequate predictions, since they do not consider the class distribution. This leads to achieving high overall accuracy, while it only reflects the accuracy of the majority class.

3 SUPPLEMENTARY FIGURES

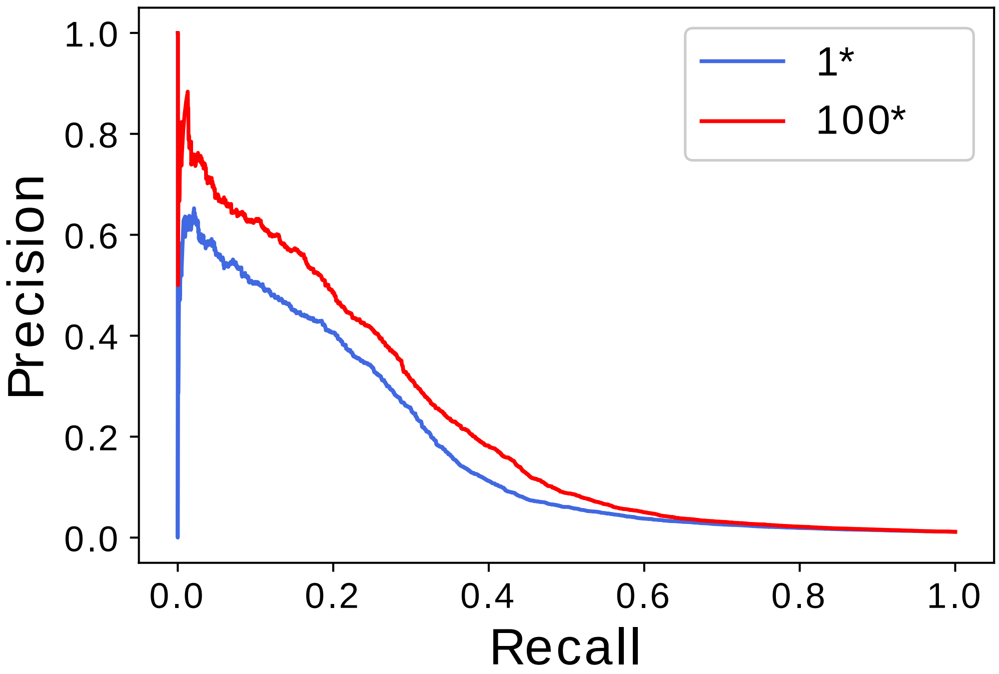

Figure 1. Precision-Recall curve for model 1* and 100* on the human chromosome X. The predictions are binned with a binning size of 600 bp. A threshold is applied to the binned prediction signal to identify predicted GSS and non-GSS containing bins. The true label of each bin as GSS or non-GSS is based on the presence or absence of a real GSS in the bin. The precision-recall curve is then obtained by changing the value of the threshold and computing the corresponding precision and recall.

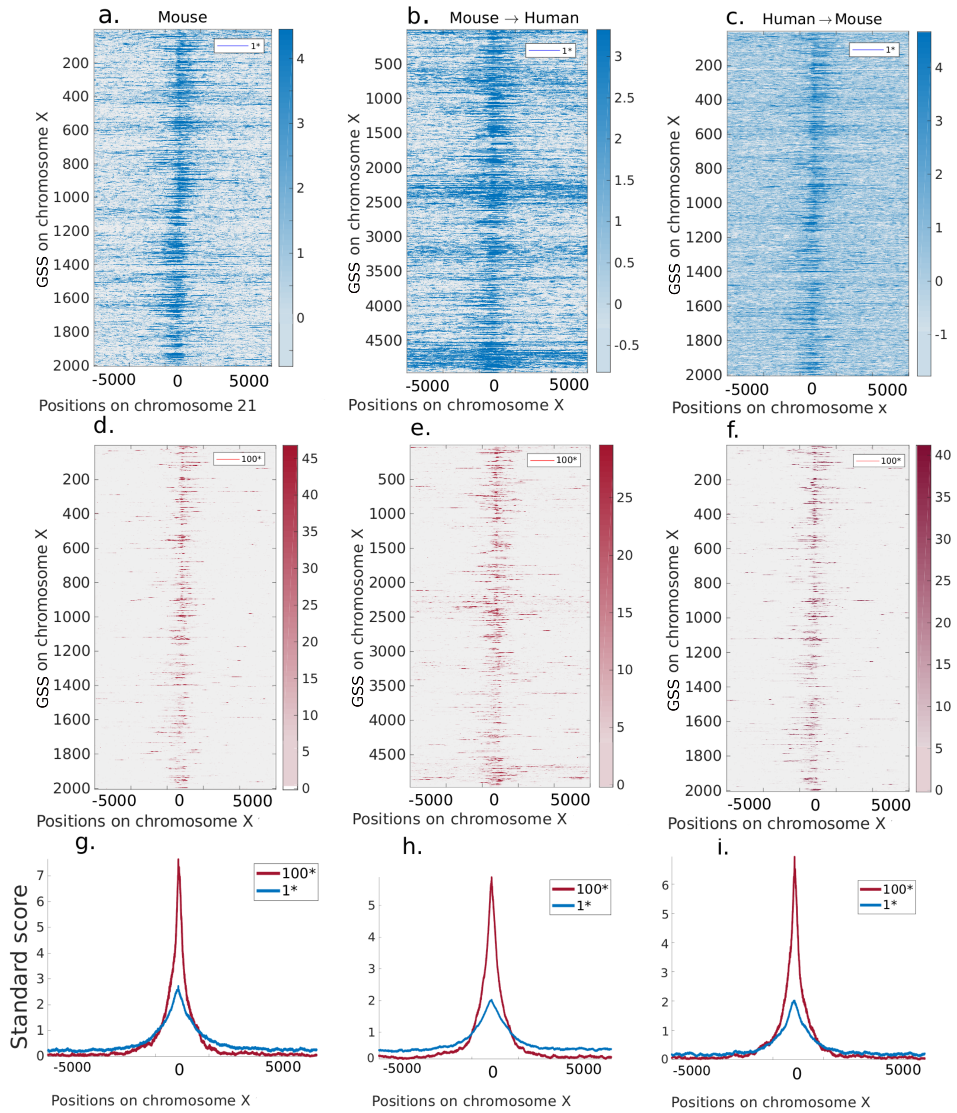

Figure 2. **Overview of human and mouse models performances over the chromosome X.** (a) and (c) Heat maps depict the standard score of the predictions for respectively the 1* model trained on mouse and applied on mouse (a), human (b) and for the 1* model trained on human and applied on mouse (c). (e) and (g) Similar to (a) and (c) with the 100* model. (h) and (j) Averaged standard score of the predictions over of the heat maps over all TSS, for the models 1* and 100* similar to (a) and (c).

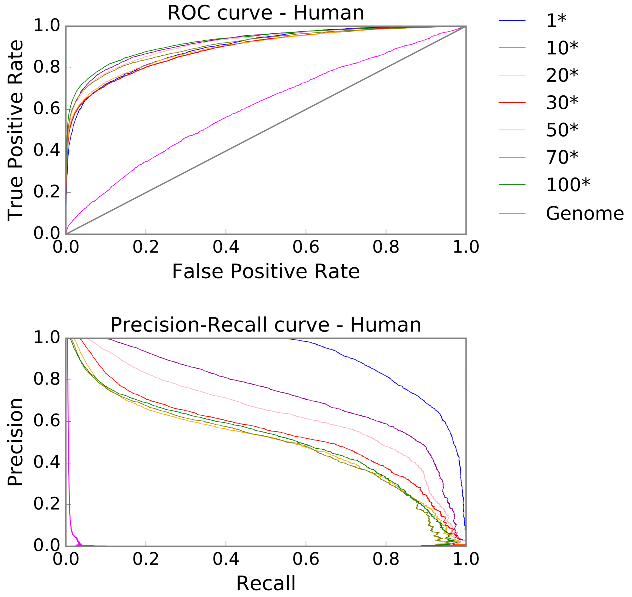

Figure 3. **Results on ROC and PR obtained over test sets.** The model 1* corresponds to a balanced dataset.

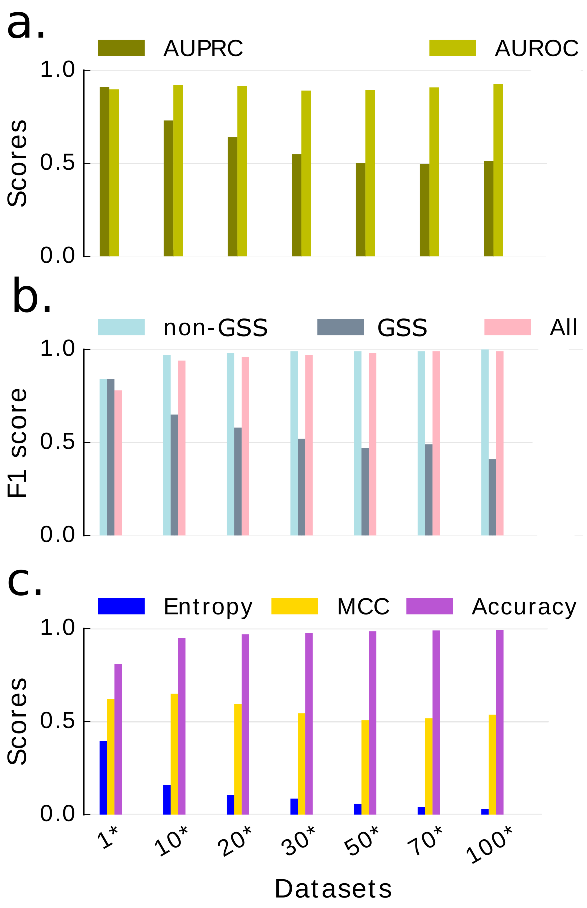

Figure 4. Evaluation the performance of CNN models for different values of Q. For each value of $Q = 1, 10, 20, 30, 50, 70 and 100$, sample sets where divided into train, validation and test sets. The results reports the performance of the models on test sets. (a) AUPRC and AUROC. (b) F1-score. (c) Variation of binary cross entropy (computed using the same weighting scheme as for training), MCC and accuracy measure.
